# Comparison of the Toponomes of Alveolar Macrophages From Wild Type and Surfactant Protein A Knockout Mice and Their Response to Infection

**DOI:** 10.3389/fimmu.2022.853611

**Published:** 2022-04-27

**Authors:** David S. Phelps, Vernon M. Chinchilli, Xuesheng Zhang, Debra Shearer, Judith Weisz, Joanna Floros

**Affiliations:** ^1^ Penn State Center for Host Defense, Inflammation, and Lung Disease (CHILD) Research and Departments of Pediatrics, The Pennsylvania State University College of Medicine, Hershey, PA, United States; ^2^ Public Health Sciences, The Pennsylvania State University College of Medicine, Hershey, PA, United States; ^3^ Obstetrics and Gynecology, The Pennsylvania State University College of Medicine, Hershey, PA, United States

**Keywords:** phagocytosis, phenotype, SP-A, surfactant, innate immunity, immunohistochemistry, immunophenotype

## Abstract

**Background:**

Surfactant protein-A (SP-A) plays a critical role in lung innate immunity by regulating alveolar macrophages (AM), expression of inflammatory mediators, and other host defense proteins. The toponome imaging system (TIS), a serial immunostainer, was used to study the AM toponome because it characterizes the localization of multiple markers and identifies marker combinations in each pixel as combinatorial molecular phenotypes (CMPs). We used TIS to study the AM toponome from wild type (WT) and SP-A knockout (KO) mice and changes following *Klebsiella pneumoniae* exposure.

**Methods:**

WT or KO mice received intratracheal *K. pneumoniae* or vehicle and AM were obtained by bronchoalveolar lavage after one hour. AM were attached to slides and underwent TIS analysis. Images were analyzed to characterize all pixels. AM CMPs from WT vehicle (n=3) and infected (n=3) mice were compared to each other and to AM from KO (n=3 vehicle; n=3 infected). Histograms provided us with a tool to summarize the representation of each marker in a set of CMPs.

**Results:**

Using the histograms and other tools we identified markers of interest and observed that: 1) Both comparisons had conserved (present in all group members) CMPs, only in vehicle AM and only in infected AM, or common to both vehicle and infected AM, (i.e., unaffected by the condition). 2) the CMP number decreased with infection in WT and KO versus vehicle controls. 3) More infection-specific CMPs in WT vs KO AM. 4) When AM from WT and KO vehicle or infected were compared, there were more unique CMPs exclusive to the KO AM. 5) All comparisons showed CMPs shared by both groups.

**Conclusions:**

The decrease of CMPs exclusive to infected AM in KO mice may underlie the observed susceptibility of KO mice to infection. However, both KO groups had more exclusive CMPs than the corresponding WT groups, perhaps indicating a vigorous effort by KO to overcome deficits in certain proteins and CMPs that are dysregulated by the absence of SP-A. Moreover, the presence of shared CMPs in the compared groups indicates that regulation of these CMPs is not dependent on either infection or the presence or absence of SP-A.

## Introduction

In a number of previous reports we have studied the effect of SP-A on the function of the alveolar macrophage (AM) (1). These studies have examined the expression of inflammatory mediators (2–5), the AM proteome (6–9), miRNome (10–12), and AM gene expression (1, 13), and more recently the AM toponome (14–16), i.e. the organization of proteins within the cells. These studies of inflammatory mediators were done, for the most part, by either comparing wild type (WT) mice or transgenic mice expressing human SP-A with SP-A knockout mice (KO), or by “rescuing” SP-A KO mice by administering exogenous SP-A. The results consistently showed differences between AM exposed to SP-A, either chronically or acutely, and AM not exposed to SP-A. The implications of these differences have been demonstrated in survival studies in a pneumonia model where mice were infected intratracheally with bacteria. SP-A KO mice showed markedly increased mortality compared to WT or “SP-A rescued” mice (17, 18). We showed that this was likely due, at least in part, to a reduction in the phagocytic index of the KO mice lacking SP-A (17).

Although we have demonstrated multiple changes in the AM in the absence of SP-A, the increased susceptibility to infection (17, 18) and reduced phagocytic efficiency (19) is likely due to many factors. The results of the proteomic studies strengthen this supposition since many proteins are affected, where some are increased and others decreased (6–9). We have previously shown that within a very short time after the introduction of bacteria to the lungs, binding of bacteria by the AM and phagocytosis of the pathogen are highly dependent on the presence of SP-A (17). The enhancement of bacterial clearance by SP-A is likely due to several mechanisms. The most proximal is the ability of SP-A to enhance phagocytosis by serving as an opsonin – binding the pathogen and enhancing its binding to cell surface receptors (20–22) . The second level is SP-A dependent regulation in the AM of various cell surface receptors involved in phagocytosis (18, 23, 24). Several of these cell surface proteins are targeted in this study, including sialoadhesin, CD206, CD44, and TLR4 (25–29). The third level is an apparent activation or boost of overall function of the AM due to SP-A (1, 22). Activation may be affected by a number of the studied proteins, including CD45, TLR4, Ly-6C, and CD200R, among others (21, 30–36). These and other proteins involved in cell-cell and cell-matrix interactions that can also influence activation were investigated in the present study.

The technology used in the present study, the Toponome Imaging System (TIS), is an ideal tool with which one may study these events because it looks at combinations of proteins, rather than individual proteins. TIS, also known as Imaging Cycler Microscopy, or Multi-epitope ligand cartography (MELC), allows one to study the toponome or the expression and/or the presence of multiple markers in their spatial networks in intact AM after different experimental manipulations (14–16, 37–39).

TIS is a robotically-driven system using immunofluorescent microscopy developed by Schubert (37–40) that involves repeated cycles of immunostaining, imaging, and photobleaching of antibodies conjugated with fluorescein isothiocyanate (FITC). A key feature of TIS is its utilization of dedicated software programs to process the captured images and construct a pixel-by-pixel map of the cells that provides information about co-localization and co-expression of proteins. This approach is significant because proteins rarely operate in isolation and their behavior may be a function of other proteins in close proximity forming multiprotein complexes. TIS generates these data by mapping each cell for the localization of multiple markers, allowing the investigator to identify and enumerate supramolecular structures formed by protein clusters, rather than simply co-localizing the proteins. These protein clusters are referred to as Combinatorial Molecular Phenotypes or CMPs, and they may identify candidate proteins that are potentially involved in important protein-protein interactions. Evidence is increasing that many proteins function as part of supramolecular complexes composed of multiple proteins working together. The scientific literature contains many examples of these interactions in pathway diagrams that illustrate various pathways such as that involving the LPS/CD14/TLR4/MyD88-mediated LPS receptor pathway (41).

In the composite images generated by TIS there are 2^n^ possible CMPs or marker combinations where n = the number of markers used (37, 40, 42, 43). CMPs are used to characterize and compare cell populations. This is important because there is evidence that various conditions result in differences in both the number and composition of CMPs; and there are conditions that may be identified by CMPs with a unique composition (37, 40, 42, 43). Thus, TIS serves as an ideal tool for addressing potential causes of alterations in AM function.

Several studies from our laboratory have employed both *in vitro* and *in vivo* assays to assess the phagocytic index of alveolar macrophages and the role of SP-A in this process (1, 44–46). The current study employed the same conditions described previously (19) for *in vivo* phagocytosis to investigate the impact of infection and/or SP-A on the AM toponome. We studied patterns of protein expression in AM harvested from lungs of WT or SP-A KO mice one hour after the intratracheal instillation of *Klebsiella pneumoniae*. The one-hour time frame used in these studies is notable in that it is unlikely that the effects we see result from new protein synthesis. Therefore, differences between vehicle-instilled and bacteria-instilled AM within a group (WT or KO) are probably a function of previously synthesized proteins moving within the cell, being secreted by the cell, or being broken down by metabolic events related to neutralizing phagocytosed bacteria. All of these events have the potential to alter the organization of proteins within the cell and change CMPs. A panel of antibodies to proteins known to have roles in AM cell-cell interactions and innate immune function was used. Our goal was to determine whether the introduction of bacteria during the time interval needed to alter the phagocytic index of the AM changed their toponome with respect to the studied group of proteins, and whether SP-A played a role, as assessed by comparison studies of the toponome of AM from WT or KO mice.

## Methods and Materials

This study used male mice on the C57BL6/J genetic background. Two strains were used – Wild Type (WT) (n=6; vehicle n=3 and infected n=3) and SP-A knockout (KO) (n=6; vehicle n=3 and infected n=3) at 8-12 weeks of age. The mice were bred in our breeding colony at the Penn State College of Medicine and raised under pathogen-free conditions or in barrier facilities with free access to food and water. Sentinel animals in the same animal rooms showed no evidence of respiratory pathogens. The Institutional Animal Care and Use Committee of the Penn State College of Medicine approved this study.

### Preparation of Bacteria

*Klebsiella pneumoniae* bacteria were obtained from the American Type Culture Collection (ATCC 43816) and cryopreserved until needed for an experiment. The bacteria were grown overnight at 37°C in TS Broth with shaking at 120 RPM for 18 hours. An aliquot was then diluted and grown for 3 hours to achieve mid-log phase growth.

### Experimental Procedure

Mice from each strain were anesthetized and received an intratracheal instillation of an aliquot of 1.2 x 10^7^ mid-log phase bacteria in 50 microliters of PBS instilled into the trachea. Control mice from each strain received vehicle (50 microliters of PBS) only. After 1 hour, AM were harvested by BAL with 5 x 500 ul of PBS, 1 mM EDTA. The infection time was determined from earlier AM phagocytosis studies (19). The cells were washed and plated, then incubated for 1 hour to allow them to attach to slides. The slides were then dried, fixed, and stored at -80°C until TIS was done.

### TIS

TIS was performed as described previously (14–16). As discussed in previous studies, the choice of antibodies was limited by the presence of artifacts in fluorescent images from some samples that may prevent the use of some of these images/markers for study. Good quality images for all 3 samples in all 4 experimental groups are needed to do a comparative CMP analysis. For our comparison study we analyzed data sets with 9 markers, although we originally performed the TIS with more markers.

### Antibody Panel

**Table 1** lists the nine antibodies or reagents we used in the image analysis, their gene names (where appropriate), their Uniprot accession numbers, the commercial source, and the catalog number of each antibody. Calibration and optimization of antibodies for the TIS procedure was done as previously described (14–16). Briefly, commercially available antibodies were tested at several dilutions. The time for incubation was held constant for all antibodies (30 minutes). The antibody concentration selected was one at which we obtained a good fluorescence signal with minimal background. We experimented with different exposure times for imaging so that we obtained good signals that were below saturation. After confirming concentration and exposure times, TIS runs were set up with the whole series of antibodies. This process was conducted for each new antibody that was added to an experiment or when there was a change in lot number for a given antibody.

**Table 1:** Markers for phagocytosis study (Wild Type) and comparison with knockout (KO).

Marker #	Marker or protein name	Accession #	Gene name	Supplier	Catalog #
1	Sialoadhesin (Siglec 1; CD169)	Q62230	Siglec1	Bioss	bs-10751R-FITC
2	CD44, Pgp-1, H_CAM, Ly-24	P15379	Cd44	BD Pharmingen	553133
3	CD200R (Mox-2; Ox-2)	O54901	Cd200r1	ThermoFisher	MA5-17984
4	CD206 (mannose receptor; C type 1)	Q61830	Mrc1	ThermoFisher	MA5-16870
5	CD45 (Receptor-type tyrosine-protein phosphatase C	P06800	Ptprc	BD Pharmingen	553080
6	CD18 (LFA-1, Mac-1) Integrin B2	P11835	Itgb2	BD Pharmingen	553292
7	Toll-like receptor 4 (TLR4), CD284	Q9QUK6	Tlr4	ThermoFisher	MA5-16212
8	Lymphocyte antigen-6c2 (Ly-6C2; Ly-6C)	P0CW03	Ly6c2 (Ly6c)	BD Pharmingen	553104
9	Phalloidon	**-**	**-**	ThermoFisher	F432

List of Markers: The markers used in this study are listed together with the accession number, gene name, supplier, and catalog number.

**Figure 1:**
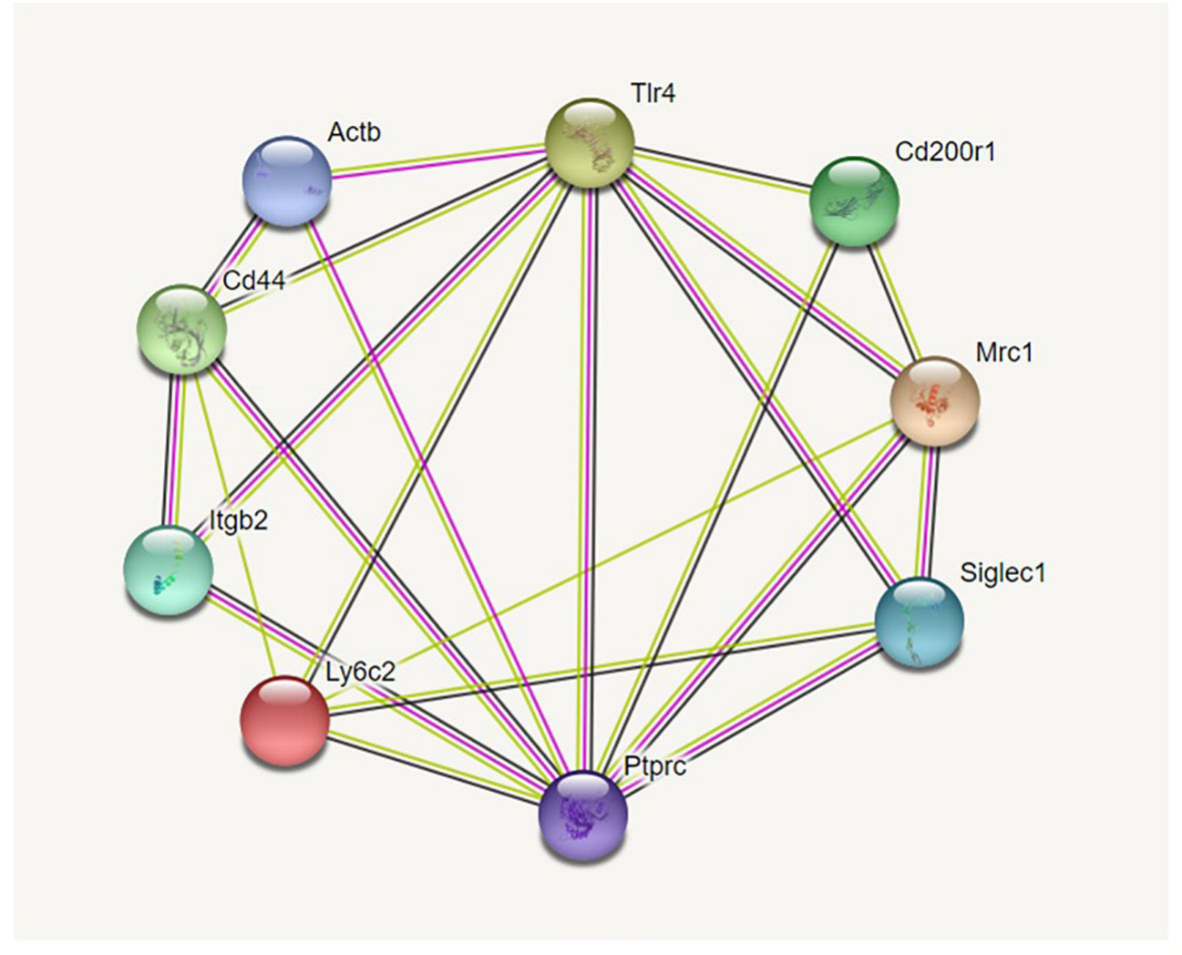
Interaction diagram from String database. **Figure 1** shows the 9-marker set of proteins we studied. Proteins more closely identified with innate immune function, and specifically phagocytosis, are grouped on the right. Proteins known for their roles in cell-cell or cell-matrix interaction/adhesion are grouped on the left. As is evident from the large number of interactions each has, TLR4 and Ptprc (CD45) are placed at the top and bottom center and play pivotal roles in both groups. The interaction diagram was generated by https://string.db.org.

Potential interactions between the markers used are shown in an interaction diagram (**Figure 1**) generated by the information present in the String database (https://string-db.org). It should be noted that we used FITC-labeled phalloidin to detect F-actin and for the interaction diagrams we used the gene symbol for beta-actin (*actb*), the main constituent of F-actin as a surrogate for phalloidin.

The selected antibodies shown here were chosen because they reliably produced good staining with a minimal number of artifacts.

### TIS Image Analysis

**Figure 2:**
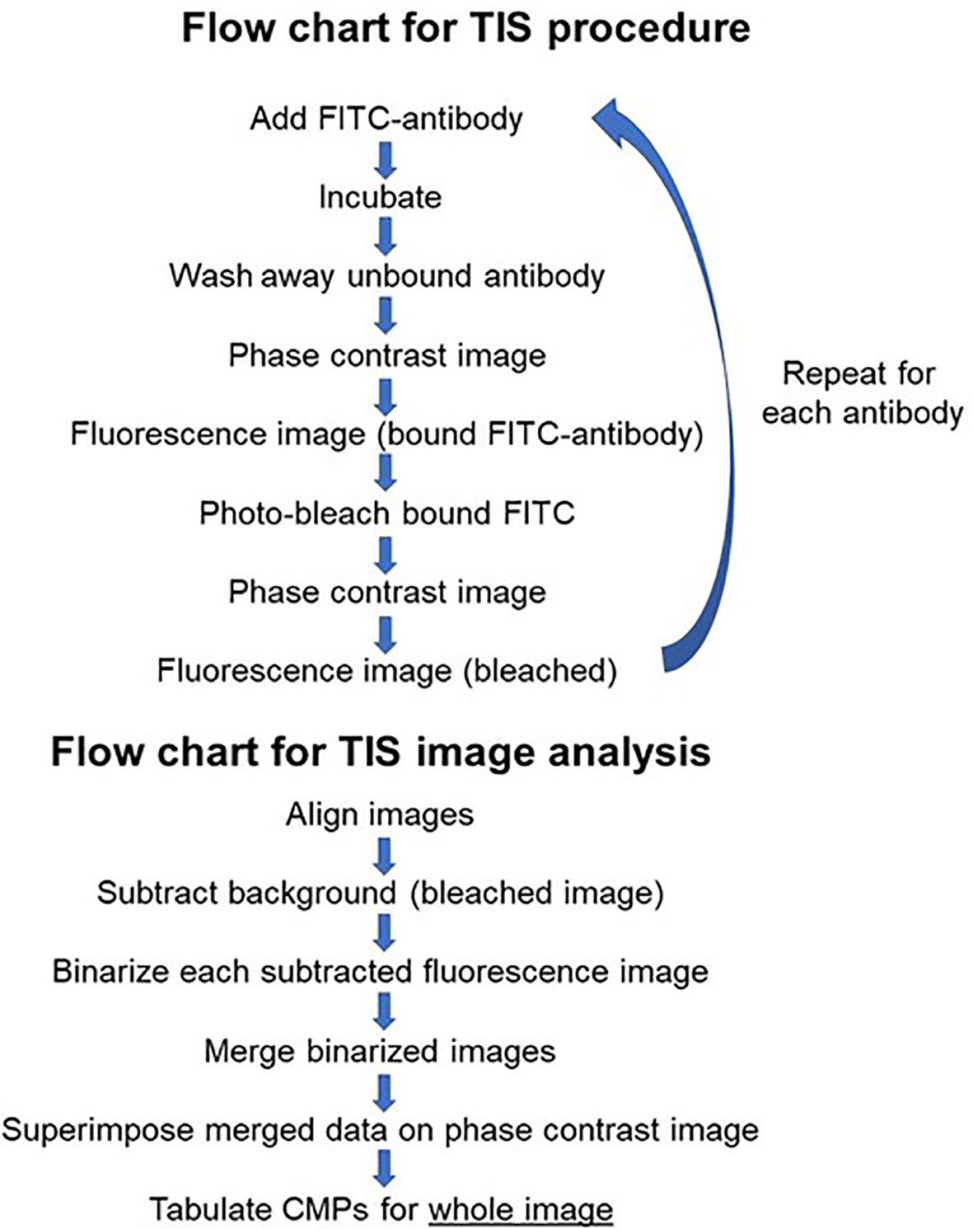
Flow chart. **Figure 2** is flow chart that describes the TIS procedure and the steps involved in the analysis of images generated by TIS.

**Figure 3:**
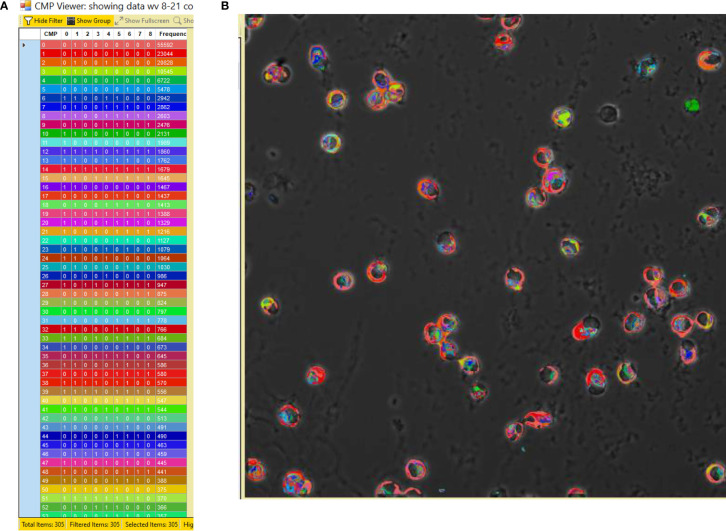
CMP data and pseudocolored image. Panel **(A)** depicts an example of a portion of a CMP data table generated by the TIS software from a combined image after binarization of each of the 9 immunostained images. The CMPs are numbered in the left-hand column and are listed in order of frequency (i.e. abundance; # of pixels) (in the right-hand column) in which that CMP is present. The intervening columns (numbered 0 to 8) indicate the absence (0) or presence (1) of each marker in a given CMP. The colors in the table **(A)** are assigned by the program and also used to pseudocolor the image from which the data were derived **(B)**.

The process employed to generate TIS data from a collection of immunofluorescent images has been described in detail in our previous publications (14–16) and is summarized in **Figure 2**. Briefly, a sample slide is subjected to repeated cycles of immunostaining with fluorescein-labeled antibodies, an image captured, the fluorescence bleached out, and the cycle repeated with the next antibody. After the immunostaining is completed, all of the resulting images are subjected to a binarization process in which a threshold level is set and areas with levels below the threshold designate the marker as absent and areas above the threshold as present. Following binarization the series of images for a given sample are combined digitally. The presence or absence of each marker is tabulated in every pixel in the image. This is denoted in data tables, such as that shown in **Figure 3**, Panel A, as (0) for absent and (1) for present. The CMPs in order of abundance are listed in the column on the far left. The columns labeled 0 to 8 list the presence or absence of each marker. The column on the far right lists the frequency (i.e. abundance; # of pixels) occupied by that CMP. Each of the tabulated CMPs is assigned a color which is used to pseudocolor the image shown in **Figure 3**, Panel B.

**Figure 4:**
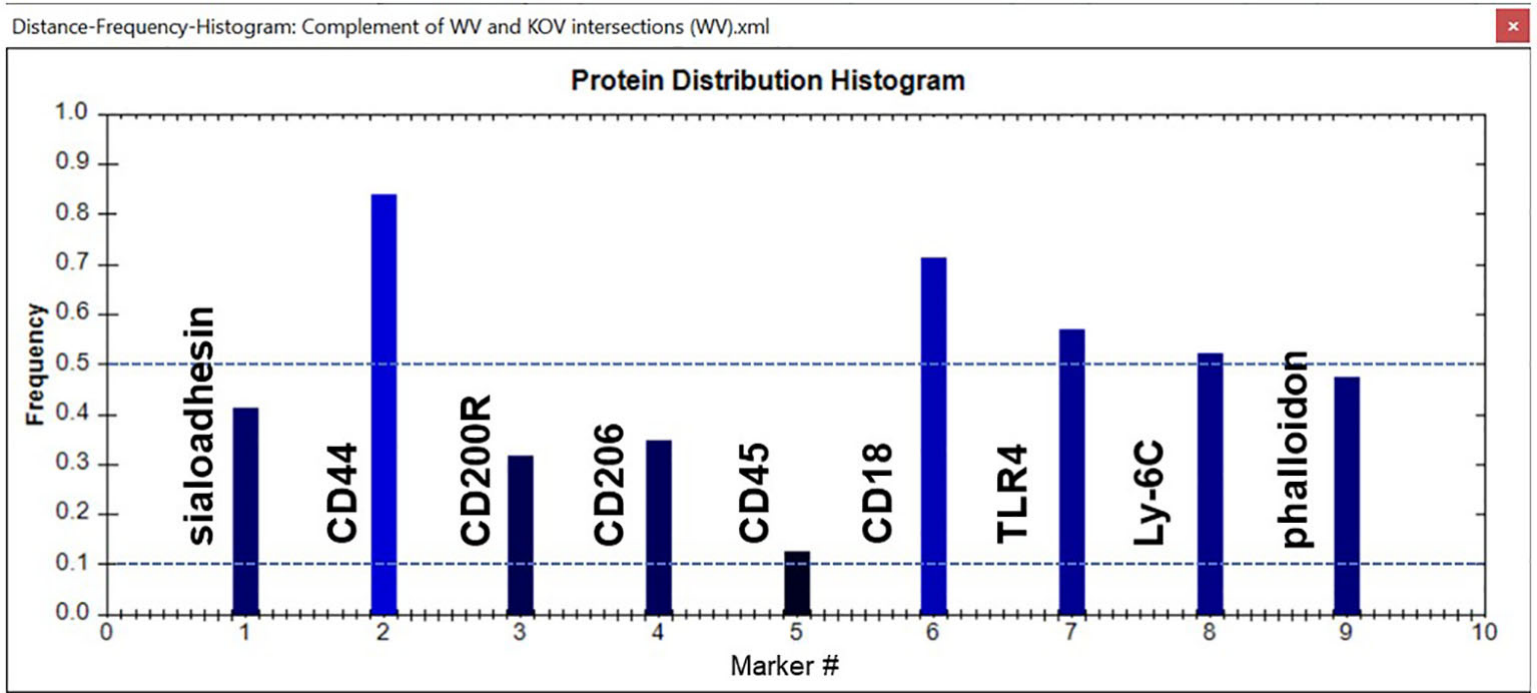
Sample histogram. An example of a CMP histogram for one of the samples is shown. Reference lines (dotted blue) have been added for frequencies of 0.1 (10%) and 0.5 (50%). The frequency shows the relative abundance of a given marker in the set of CMPs used to generate the histogram. In this case Marker #2 (CD44) is found in 83% of the CMPs, whereas Marker #4 (CD206) is only found in 12% of the graphed CMPs. The names of the markers represented by each bar are shown.

In this study image analysis was restricted to whole images. In some samples, cell aggregation prevented us from delineating and analyzing separate cells. We used several tools within the TIS software to analyze the data in the images from the various experimental groups. One tool is a histogram that provides a snapshot of the group of CMPs being analyzed. An example is shown in **Figure 4**. Each marker is represented as a bar rising from the x-axis. The y-axis shows the frequency of each marker in all the CMPs being graphed. Note that in most cases this value would differ from the absolute amount of each marker because the CMPs themselves may vary considerably in terms of their abundance or the number of pixels in which they are found. In this example marker #2 (CD44) is found in about 83% of the CMPs and marker #5 (CD45) is found in about 12%.

**Figure 5:**
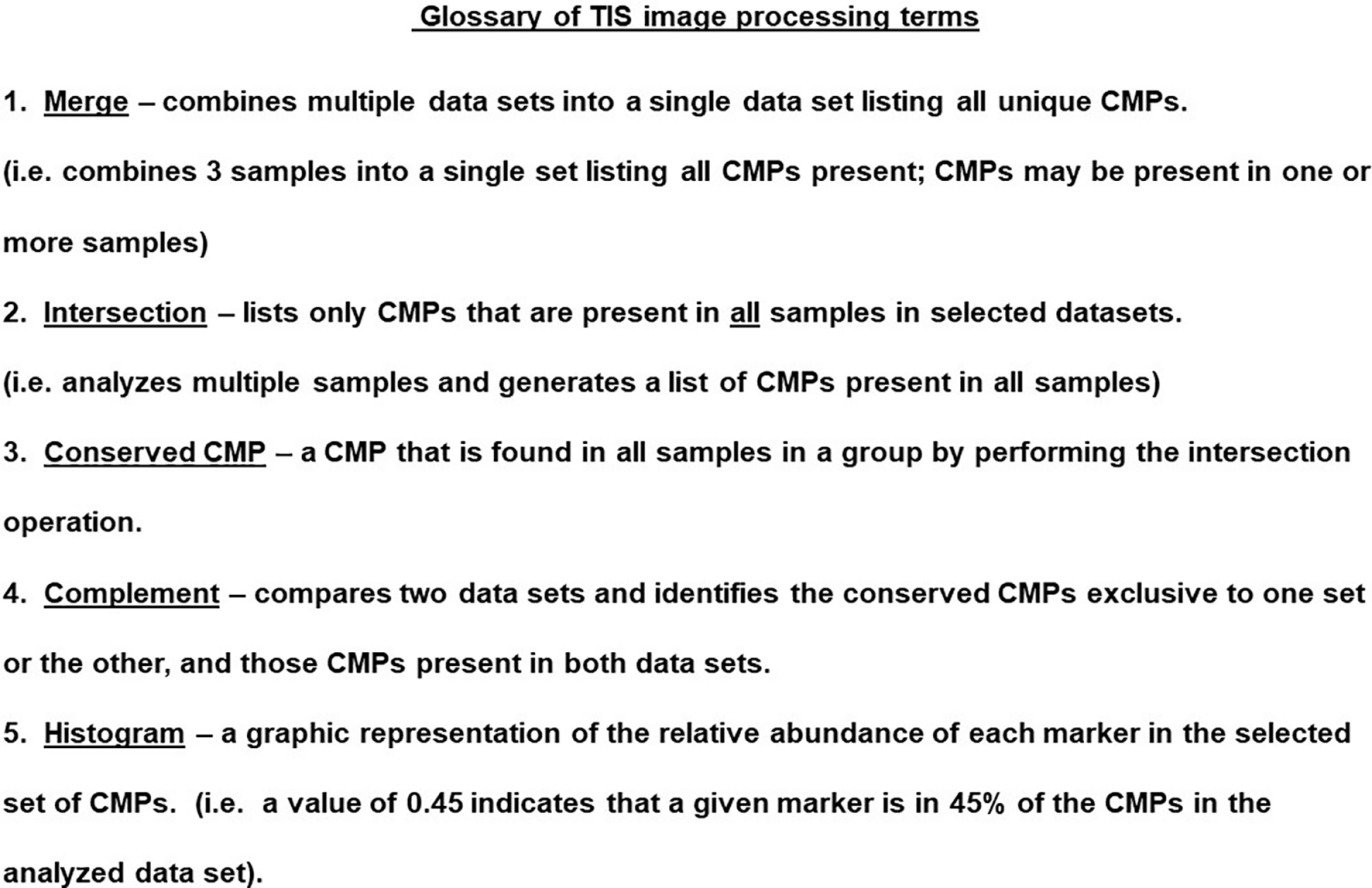
Glossary of TIS terms. The TIS software enables probing the CMP data in a variety of ways. Several terms and the operations are listed and described in this figure.

The TIS software also allowed us to analyze the data in a number of ways that are subsequently shown in the histograms. These operations are summarized in **Figure 5** and include the ability to merge datasets, to find the intersection of multiple datasets, and determine the CMPs unique to one dataset compared to another (i.e. complement). These operations allowed us to identify conserved CMPs (i.e. CMPs present in multiple datasets).

## Results

### Marker Interactions

Using the STRING database (https://string-db.org) we constructed diagrams of potential interactions between the proteins listed in **Table 1** from the information in the STRING database. The selected markers were proteins known to be important in AM function with a number of “potential” interactions among themselves. Two proteins, TLR4 and CD45 (Ptprc), exhibit interactions with the other eight proteins included in **Figure 1**. The interaction diagram has been constructed so that proteins involved in cell-cell interactions and cell adhesion are grouped on the left side of the diagram and proteins implicated in host defense function are on the right side. However, as the diagram illustrates these are not clear divisions and there are numerous interactions among the proteins. Five of the nine molecules potentially interact with more than half of the other molecules. The large number of documented interactions in various systems depicted by the diagram shows the relevance of these proteins and their likely importance in macrophage function.

### Effect of Infection on the Toponomes of WT and SP-A KO AM

In order to assess how infection with *Klebsiella pneumoniae* affects the AM toponome and the influence of SP-A, we compared the response of AM to treatment with vehicle or bacteria in WT AM and SP-A KO AM. To do this it was necessary to have immunofluorescent images in all three samples that were free of artifacts for all four groups. The resultant set of 108 images included nine markers. **Figure 3**, Panel B is an example of a representative AM sample after pseudocoloring by the TIS software.

#### Comparison of WT Vehicle vs. WT Infected Toponomes

The six WT samples in this analysis (n=3 vehicle; n=3 infected) had an average of 263 CMPs (range = 163 to 343). The details for each group are listed in **Table 2**, which shows the number of CMPs in each sample and their average. When the three WT vehicle (WV) samples are merged there are a total of 392 unique CMPs among the samples in the group. Determining the intersection of the three samples in each group provides a tabulation of CMPs that are present in all three samples in the group. We refer to these as “conserved” CMPs because they are found in all samples in a group. In the case of the WT vehicle group the intersection is comprised of 176 CMPs.

**Table 2:** Summary of CMPs in original samples and created data sets.

	WT-vehicle	WT-infected	KO-vehicle	KO-infected
Sample A	305	163	347	270
Sample B	211	254	294	214
Sample C	343	300	239	271
Average	286	239	293	252
Intersection	176	102	207	123
Merged (A,B,C)	392	392	449	390
Merged (veh+inf)	449		476	

The four columns list data for each experimental group. The three samples (A, B, C) for each group are listed along with their average. The samples were then analyzed for their intersection (CMPs common to all three samples) (see **Table 2**) and the three samples were merged to obtain the total number of unique CMPs in each group of three samples. In the bottom row of the table is the number of unique CMPs found in the merged wild type group and the KO group.

The second column of **Table 2** lists the CMP numbers for the WT infected (WI) group. The number of CMPs from the WT vehicle and WT infected samples did not differ significantly from one another. Merging the three WT infected samples also resulted in a set of 392 unique CMPs. The intersection of the WT infected group consisted of 102 CMPs. The data set formed by merging all of the vehicle samples with all of the infected samples contained a total of 449 unique CMPs. The theoretical maximum number of CMPs in a 9-marker study is 2^9^ or 512 CMPs. The last two columns in **Table 2** show comparable data for the KO-vehicle and KO-infected samples and are discussed below in the next section.

**Figure 6:**
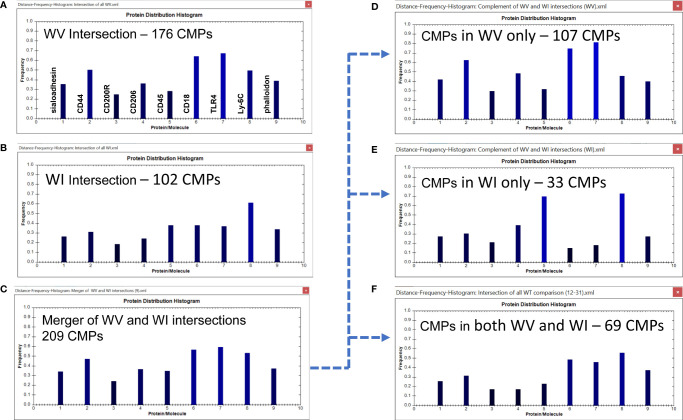
CMP comparison of wild type AM with nine markers. The names of the proteins represented by each histogram bar are shown in Panel **(A)**. The same order applies to all other histogram panels in this and subsequent figures. Panels **(A, B)** show the intersection of the three wild type vehicle and three wild type infected samples. The intersection feature identifies the “conserved” CMPs present in all three samples of each group. Panel **(C)** depicts the merged results of Panels **(A, B)** and shows the characteristics of the 209 CMPs that are conserved in the vehicle and/or infected groups. Panels **(D–F)** use the complement feature of the TIS software to identify from the merged 209 CMPs, CMPs present in vehicle AM only **(D)**, infected AM only **(E)**, or common to both groups **(F)**. The arrow originating at Panel **(C)** and terminating at Panels **(D–F)** illustrates this process.

We next looked at the histograms depicting intersection of all samples in the two groups which shows the marker composition of all “conserved” CMPs that were present in every sample (3-out-of 3) of each group. These and subsequent data sets are characterized in histograms, such as the one described above (**Figure 4**); these show the relative frequency of each marker in the sampled CMPs. As stated above, we identified 176 CMPs that were present in every WT vehicle sample (**Figure 6A**) and 102 CMPs that were present in the three WT infected samples (**Figure 6B**). The observed reduction (**Figure 6B**) in the number of CMPs after infection (102 in WI vs 176 in WV, **Figure 6A**) may indicate that exposure to bacteria results in a loss of phenotypic diversity as the AM mobilize to deal with the infectious threat. Merging these two sets (vehicle and infected) of conserved CMPs revealed that in the WT AM there were 209 unique CMPs that were conserved in at least one sample (**Figure 6C**).

When this set of 209 conserved CMPs was subjected to the complement procedure we were able to determine how many of the CMPs present in all three samples were: a) exclusive to the WT vehicle group (**Figure 6D**); b) exclusive to the WT infected group (**Figure 6E**) or; c) common to both groups (i.e. present in all three samples of both groups) (**Figure 6F**). The broken arrow originating at **Figure 6C** and terminating at **Figures 6D**–**F** illustrates this process. These conserved CMPs were mostly found in the vehicle group (n=107) (**Figure 6D**) and may be viewed as being characteristic of the normal AM. There are 33 CMPs that are exclusive to the infected group (**Figure 6E**) and these are probably triggered by the bacterial exposure and are likely to be involved in host defense processes against the bacteria. Finally, there are 69 conserved CMPs that are common to both groups (**Figure 6F**) and these may represent housekeeping functions since they are present in both groups and not affected by infection.

Using all of the CMP data the mean levels of each marker were calculated in each of the three samples/group. When these were compared using the Wilcoxon Rank Sum test the WT vehicle and WT infected data sets were found to be significantly different (p=0.0424).

Some of the features of these groups offer some insight into differences resulting from the exposure to bacteria. For example, the conserved CMPs found in the vehicle-treated AM (**Figure 6A**) generated a histogram that was quite different from that in the WT infected AM (**Figure 6B**). WT vehicle AM were characterized by high levels of CD44, CD18, and TLR4 (**Figure 6A**), whereas most of those markers were at lower levels in the group of conserved CMPs found in the infected AM (**Figure 6B**). The overall differences between vehicle-treated and infected were more pronounced in the histograms of the group-exclusive CMPs (**Figures 6D, E**). As with the whole group of conserved CMPs the vehicle treated AM had higher levels of CD44, CD18, and TLR4, but it is also notable that levels of CD45 and Ly-6C were markedly lower in the WT vehicle AM than in their infected counterparts. These differences are likely to be part of the AM response to bacterial challenge.

It is also of interest to note that the overall profile of the histogram bars in the common CMPs (**Figure 6F**) for many of the markers was more similar to the profile of CMPs exclusive to the vehicle-treated group (**Figure 6D**), albeit at lower frequencies. The profile of CMPs exclusive to the infected AM (**Figure 6E**) differs from the subset of CMPs common to both groups (**Figure 6F**) in terms of many of the proteins.

#### Comparison of SP-A KO Vehicle vs. SP-A KO Infected

An analysis similar to the one described above for the WT was performed on the AM from KO mice. The six samples in this analysis (three vehicle; three infected) had an average of 273 CMPs (range = 214 to 347). The CMP data for the KO vehicle (KOV) and KO infected (KOI) groups are listed in **Table 2**. When the data for the three KO samples in each group were merged, a total of 449 unique CMPs were identified in the vehicle AM and 390 in the infected. A merging of these two sets (vehicle and infected) resulted in 476 unique CMPs.

**Figure 7:**
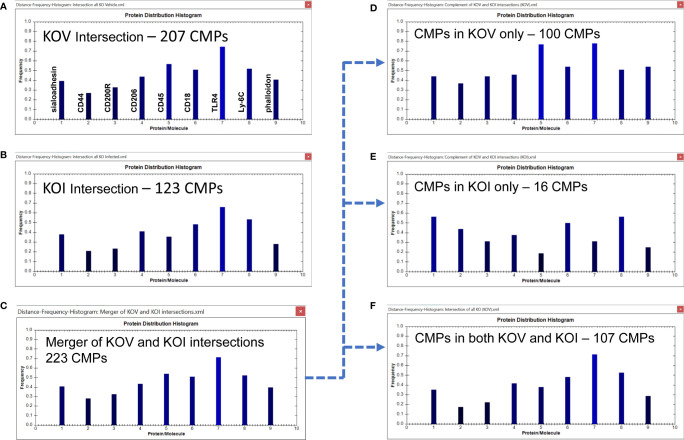
CMP comparison of KO AM. As described in **Figure 6**, Panels **(A, B)** show the intersection of the three KO vehicle and three KO infected samples. Panel **(C)** depicts the merger of Panels **(A, B)** and shows the characteristics of the 223 CMPs that are conserved in the vehicle and/or infected groups. Panels **(D–F)** use the complement feature of the TIS software to identify from the merged 223 CMPs, CMPs present in KO vehicle AM only **(D)**, KO infected AM only **(E)**, or common to both KO groups **(F)**. The arrow originating at Panel **(C)** and terminating at Panels **(D–F)** illustrates this process.

Using the TIS software we obtained histograms for the intersection of all samples in the two groups; this tabulates all “conserved” CMPs that were present in every sample (3-out-of-3) of each group. We identified 207 (out of the total of 449) CMPs to be present in all three KO vehicle samples (**Figure 7A**) and 123 CMPs (out of 390) in the three KO infected samples (**Figure 7B**). This reduction in conserved CMPs after infection mirrors what we observed in the WT AM and provides another example in support of our conjecture that exposure to bacteria results in a loss of phenotypic diversity as the AM mobilize to deal with the potential infection.

Merging these two sets of intersecting CMPs (**Figures 7A, B**) revealed that they comprised of 223 unique CMPs that were conserved in at least one of the KO groups (**Figure 7C**). As with the WT data, when we subjected this set of 223 CMPs to the complement procedure with the TIS software, we found that there were 100 conserved CMPs (i.e. present in all three samples) that were: a) exclusive to the vehicle-treated group (**Figure 7D**); b) 16 conserved CMPs that were exclusive to the infected group (**Figure 7E**); and c) 107 CMPs that were common to both groups (**Figure 7F**). As in **Figure 6** the arrow originating at **Figure 7C** and terminating at **Figures 7D**–**F** illustrates this process. As described above, we broadly characterize these three categories as baseline, infection-related, and housekeeping functions, respectively. When the KO vehicle and KO infected data sets were compared using the Wilcoxon Rank Sum test they approached (p=0.076), but did not reach significance. The lack of significance in this comparison is probably a function of the blunted response to bacteria by the KO AM.

We observed one major difference from the WT comparison when we compare the histograms of the conserved CMPs in KOV and KOI AM (**Figures 7A, B**). The two histograms in KO are similar to one another, although CD45 is substantially greater in the KOV AM, and TLR4 and phalloidin had slightly higher frequencies in the KOV than in KOI. This overall impression differs from that of the corresponding histograms in the WT AM (**Figures 6A, B**) where more differences in both magnitude and pattern of the histogram bars were observed.

The differences between groups are much more pronounced in the histograms of the CMPs exclusive to the KO vehicle-treated and KO infected groups (**Figures 7D, E**). In these histograms the frequencies of CD45 and TLR4, and to a lesser extent phalloidin, are higher in the KOV AM. An opposite trend was observed for CD45 in the WT AM (**Figures 6D, E**) where CD45 was much higher in the infected AM. TLR4 followed the same pattern in both WT and KO AM with much higher levels in the vehicle-treated versus infected. Whether this difference, and the lack of differences in CD44, CD18, and Ly-6C that we observed in WT (**Figures 6D, E**) are responsible for the increased susceptibility to infection in KO mice remains to be determined.

### Effect of SP-A on the AM Toponome of Vehicle and Infected Groups

**Figure 8:**
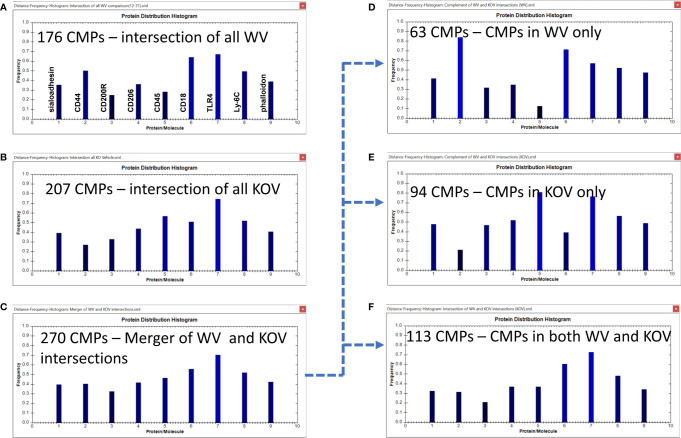
CMP comparison of wild type vehicle (WV) and KO vehicle (KOV) samples. Panels **(A, B)** show the intersection of the three wild type vehicle and three KO vehicle samples. Panel **(C)** shows the results when the data in Panels **(A, B)** are merged and shows the characteristics of the 270 CMPs that are conserved in vehicle-treated WT and/or KO groups. Panels **(D–F)** use the complement feature of the TIS software to identify, from the 270 merged CMPs, CMPs present in wild type vehicle AM only **(D)**, KO vehicle AM only **(E)**, or common to both vehicle groups **(F)**. The arrow originating at Panel **(C)** and terminating at Panels, **(D–F)** illustrates this process.

**Figure 9:**
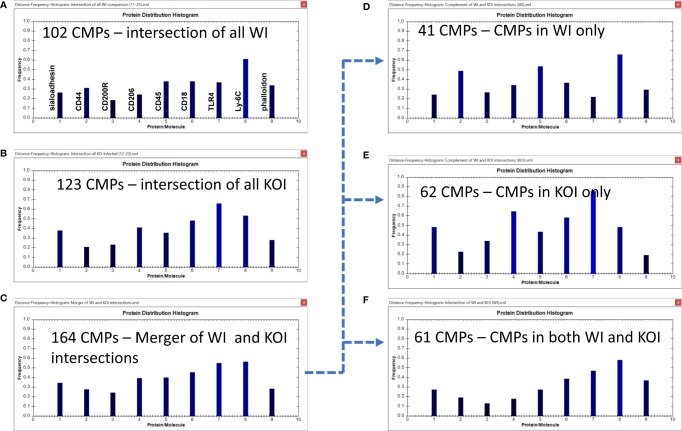
CMP comparison of wild type infected (WI) and KO infected (KOI) AM. Panels **(A, B)** show the intersection of the three wild type infected and three KO infected samples. Panel **(C)** shows the results when the data in Panels **(A, B)** are merged and shows the characteristics of the 164 CMPs that are conserved in the WT and/or KO infected groups. Panels **(D–F)** use the complement feature of the TIS software to identify, from the 164 merged CMPs, CMPs present in wild type infected AM only **(D)**, KO infected AM only **(E)**, or common to both vehicle groups **(F)**. The arrow originating at Panel **(C)** and terminating at Panels **(D–F)** illustrates this process.

We next compared the WT and KO vehicle groups to each other (**Figure 8**) and the WT and KO infected groups to each other (**Figure 9**) to better assess the role of SP-A on the regulation of the AM toponome in the presence or absence of SP-A.

#### Vehicle Groups

As shown previously the WV samples had 176 conserved (i.e. present in all three samples) CMPs (**Figures 6A**, **8A**) and the KOV samples had 207 conserved CMPS (**Figures 7A**, **8B**). For this analysis we compared the two (WT and KO) vehicle data sets. In the conserved CMPs from the two vehicle groups some slight differences in marker content were apparent (compare 8A and 8B). These included a somewhat higher frequency of CMPs containing CD44 and CD18, and a lower frequency of those containing CD45 (marker 5) in WV versus KOV (compare **Figures 8A, B**). Following merger of the data for the three samples in each vehicle group, we obtained 270 unique CMPs (**Figure 8C**). When the complement procedure was applied to these 270 conserved CMPs, it showed: a) 63 that were exclusive to the WV group only (**Figure 8D**); b) 94 CMPs that were present in KOV only (**Figure 8E**); and c) 113 CMPs were common to both groups (**Figure 8F**). As in **Figure 6** the arrow originating at **Figure 8C** and terminating at **Figures 8D**–**F** illustrates this process. These three sets of CMPs are likely to correspond to CMPs that are SP-A dependent, those that result from the absence of SP-A, and those whose expression in independent of the presence of SP-A, respectively. The histograms show that the relative abundance of the markers in the exclusive groups (**Figures 8D, E**) was similar to that described above for the corresponding conserved CMP sets defined by the intersection of the three samples in each group (**Figures 8A, B**), respectively, but more accentuated. Namely, CD44 and CD18 were much increased in WV vs KOV, and CD45 was much lower in WV than in KOV.

A comparison of these two groups (WV and KOV) with the Wilcoxon Rank Sum test, as done above with the other comparisons, found no difference between the WV and KOV groups (p=0.711).

#### Infected Groups

The WI samples had 102 conserved CMPs (**Figures 6B**, **9A**) and the KOI had 123 conserved CMPs in KOI samples (**Figures 7B**, **9B**). Note that, as discussed above, there are substantially fewer conserved CMPs in the infected AM than in the vehicle-treated AM (**Figure 8**). When we identified conserved CMPs exclusive to either WI or KOI we found: a) 41 CMPs that were only in WI samples (**Figure 9D**); b) 62 only in KOI samples (**Figure 9E**); and c) 61 CMPs that were common to both groups (**Figure 9F**). As in **Figure 6** the arrow originating at **Figure 9C** and terminating at **Figures 9D–F** illustrates this process. This categorization of these groups, as described above, includes CMPs dependent on the presence of SP-A, the absence of SP-A, and independent of SP-A, respectively. Although the marker differences might have been rather subtle in the histograms for all conserved CMPs in each group (**Figures 9A, B**), the differences in marker frequencies were more prominent when the histograms depicting the profiles of conserved CMPs found only in one group or the other were compared (**Figures 9D, E**). In WI versus KOI the histograms showed that the frequency of CD44 and Ly-6C (markers 2 and 8) in conserved CMPs was higher, and that of sialoadhesin, CD206, CD18, and TLR4 was lower (see markers 1, 4, 6, and 7) in **Figures 9D, E**). The differences in so many markers involved in phagocytosis and AM activation may be part of the reason for the compromised host defense function in KO mice. However, despite these differences when a statistical comparison involving all marker means was subjected to the Wilcoxon Rank Sum test, no significant difference was found (p=0.518).

## Discussion

Numerous studies have investigated the effects of SP-A on various aspects of innate immune function (17, 18, 47). These effects are demonstrated in studies from our laboratory and others that show the vulnerability to infection of mice lacking SP-A (17, 18). Although there are many different ways that host defense in the lung can be influenced, arguably one of the most important is at the point where an infectious organism comes into contact with the alveolar macrophage, the sentinel cell of lung innate immune function. This event triggers a cascade of downstream events that profoundly influence the ability of a cell and eventually of the organism to deal with a threat from a pathogen and survive. As mentioned earlier, the fact that this study employs a relatively short time frame (i.e. harvest of AM 1 hr after instillation of vehicle or bacteria) lessens the likelihood that we are looking at new protein synthetic events. Thus, the changes we observed are probably the result of the re-organization of existing proteins into new CMPs, making TIS an ideal tool to study this.

In previous TIS studies of the AM we have described a way to assess the phenotypic consistency or diversity of the AM in an experimental group by determining whether specific CMPs are consistently expressed or “conserved” among members of that experimental group (14–16). Given the large number of potential protein combinations or CMPs that could be identified with TIS (i.e. 2^n^ where n = number of markers tested) (14–16) it is likely that the presence of specific CMPs in multiple samples is not random, but rather due to regulatory influences characteristic of that experimental group. Although in this study we were unable to characterize individual cells, the histograms provided us with a tool to summarize the marker content of a group of CMPs and identify markers whose variability could be important. Our assumption is that these conserved CMPs mediate critical functions and/or are characteristic of the presence or absence of a key regulator, such as SP-A. In the present study, we addressed this problem by analyzing our data sets in two ways. In the first part of the study we did direct comparisons of AM treated with vehicle to infected AM to assess the effects of infection on the AM toponome in the presence or absence of SP-A. In the second part of the study we compared: a) vehicle-treated WT and KO to assess the role of SP-A on the baseline AM proteome (i.e. unprovoked AM toponome); and b) infected WT and KO AM to assess, *via* this comparison, the role of SP-A on the AM toponome in response to infection.

### Impact of Infection on the AM Toponome in the Presence or Absence of SP-A

In the first set of comparisons, which consisted of CMPs exclusive to either vehicle or infected AM, we observed that there were far more conserved CMPs in the vehicle group than in the infected group. We speculate that this reflected the fact that normally populations of AM have the potential to differentiate along multiple pathways, as predicted by the heterogeneity that typically characterizes AM populations (14–16, 48) and that the larger number of conserved CMPs in the vehicle-treated cells reflect AM pluripotentiality. However, when a challenge occurs, such as infection, many of these conserved CMPs reorganize into a smaller group of CMPs that are focused on dealing with the pathogen. Thus, a decrease in the number of conserved CMPs is observed as the AM redirects its proteins to focus on innate immune defenses against the bacteria rather than maintaining readiness to proceed down many potential pathways. Moreover, the general reorganization of CMPs to a smaller group of CMPs in response to infection may be independent of SP-A, as both WT and KO were identified with a reduced number of CMPs when each was compared to its corresponding vehicle group. However, the specific nature of CMPs varies as differences between the CMP histograms of the WT vehicle and WT infected AM as well as KO vehicle and KO infected were observed. These may reflect qualitative differences between WT and KO in the attempt to combat infection. Furthermore, although the number of CMPs in WT vehicle and KO vehicle didn’t differ significantly (107 vs 100), the number of CMPs in their corresponding infected group was quite different. The KO infected had about half as many exclusive CMPs (i.e. CMPs found only in that group) compared to WT infected (16 vs 33). This may indicate that the KO AM is only capable of invoking a limited number of pathways to combat infection, because: a) its baseline or readiness of AM is compromised in the absence of SP-A; b) the AM is unable to recognize and ingest a pathogen and initiate a successful response in the absence of SP-A; or c) both of these possibilities are contributing to the poorer outcome of KO animals in response to infection.

Of interest, in the absence of SP-A, the KO AM is not dormant and it still generates unique CMPs either in response to vehicle or infection. However, the relatively small number of infection-specific CMPs in the KOI AM versus the KOV, and the presence of twice as many infection-specific CMPs in the WT infected AM versus the KOI, indicate a greater capacity for WT to respond to an infectious challenge and a potential inadequacy of the KO to respond and successfully overcome the challenge as we have shown by survival studies (12, 17, 18), Moreover, these CMPs constitute only a fraction of the AM’s repertoire and there are likely to be other subsets, when more or other markers are used, enabling other macrophage functions to occur, such as responses to tissue damage, toxins, particulates, etc. In summary, infection appears to cause a shift of proteins into CMPs needed to respond to the insult. The importance of SP-A in this function is demonstrated by the more vigorous response in the WT AM.

### Impact of SP-A on the AM Toponome

In our second set of comparisons we shifted the focus to the effects of SP-A on the AM toponome under baseline conditions and in response to infection by studying vehicle-treated AM from WT and KO mice and then WT and KO AM from infected mice. Knockout mice lacking a functional SP-A gene have been studied for many years (47). Although there is no major apparent phenotypic difference between the two types of mice (WT and KO) under normal, unchallenged conditions (49), our study utilizing TIS and examining expression and localization of nine markers with important roles in host defense function shows that the potential of AM from these two groups is not equivalent. Even though in the present study the number of CMPs didn’t seem to differ significantly in AM from WT and KO vehicle (i.e. at baseline) the quality of these differs, as shown by the histograms. The histograms provided us with a tool to identify markers that differed between groups of CMPs. Proteomic studies also have shown that the AM proteome from KO also differs from that of the WT and that upon SP-A rescue the KO proteome becomes similar to that of the WT (6). Although the present findings are consistent with previous published studies, these further indicate that AM protein clusters or combinatorial molecular phenotypes (i.e, CMPs), rather than single protein levels, may underlie the previously observed differences at either baseline (15) or in response to infection (17). For the latter, both quantitative (i.e. the number of CMPs) and qualitative CMPs differences between WT and KO were observed. These CMPs may, in part, be SP-A-dependent and responsible for the poorer outcome in KO of either AM function in terms of their phagocytic index (17) or other AM functions (47, 49).

In the absence of an infectious challenge, the marked differences observed in the histograms in the CMP frequency of CD44, CD45, and CD18 between WT and KO may be predictive of potential host defense deficits in the AM given the importance of these proteins to host defense function (1, 30–32, 35, 36, 50). These differences were confirmed when we compared the two groups after an infectious challenge with *K. pneumoniae*, as differences in the infected groups in the same markers were observed as those seen in the vehicle-treated groups. However, in response to infection, marked differences were also observed in the CMP frequency of CD206, TLR4 and Ly-6C, all of which have important host defense functions (27, 28, 33, 34, 51).

The comparisons of the histograms from the two vehicle AM sets (**Figure 8**) and the two infected AM sets (**Figure 9**) offer some additional insight into the importance of SP-A. In previously published BAL proteomic studies (7, 52) we have observed that KO AM may overcompensate for deficits resulting from the lack of SP-A. The higher numbers of conserved CMPs in the present study in the KO groups versus the WT may be a manifestation of this. Not only are there overall more conserved CMPs in the two KO groups (vehicle and infected) but the sets of CMPs exclusive to KOV and KOI AM (**Figures 8E** and **9E**) are ~50% greater than the corresponding WT groups. However, despite the increased number of CMPs, the host defense function of the KO AM is inadequate, probably due to dysregulation of some important proteins, as shown by differences in the histograms of the two infected groups. CD45 (marker 5), a marker that varies considerably in the histograms from different groups, is a good example of the potential loss of mechanisms that may enable the formation of CMPs. The cellular localization of CD45 is dynamic and its distribution can be altered by conditions (30). Its baseline (vehicle) levels are low in WT AM and high in KO AM and just the reverse with infection, implying that its incorporation into CMPs varies with SP-A status. CD45 has many different forms and many different ligands and is involved in numerous aspects of immune cell function (30–32, 50). Given the multiple roles of SP-A in immune cell regulation it is likely that SP-A is involved in CD45’s regulation or the regulation of its ligands and thus further investigation in this area is needed.

### Common CMPs

There are also a number of conserved CMPs that are common to both vehicle and infected groups. The CMPs common to both vehicle and infected AM may be important for baseline or “housekeeping” functions in the AM. This notion is further supported by the observation that common CMPs exist in both WT vehicle and KO vehicle AM, as well as in WT infected and KO infected. These may, in part, provide a basis for the fact that KO mice, in the absence of infectious or inflammatory challenge, exhibit no major phenotypic differences from WT (49), although differences in the AM proteome between KO and WT have been observed previously (7). However, their histograms in the present study share more similarities than differences indicating that the AM baseline phenotype or its “prepared” state for future host defense is similar, but not identical between WT and KO. The common CMPs between WT and KO may be thought of as SP-A-independent. CMPs unique to WT and absent in KO at either baseline or in response to infection may be thought of as SP-A-dependent and it is likely that at least some of these play a role in mounting an effective response to infection.

In summary, using TIS and a set of nine macrophage markers we showed: a) sets of CMPs present in both WT and KO AM whose expression is independent of SP-A; b) CMP differences between vehicle-treated WT and KO AM probably due to the presence or absence of SP-A; c) a decrease in the total number of conserved CMPs in the infected AM of both groups vs vehicle, possibly due to the mobilization of proteins into specific CMPs needed to deal with infection; and d) after an infectious challenge a number of differences in the relative CMP frequencies of different proteins existed between CMPs exclusive to the challenged AM from WT and KO AM. For the latter we postulate: 1) that these differences are responsible for the effective and ineffective response to infection of WT and KO AM, respectively, as shown by survival studies (17, 18); and 2) that these would largely be reversed by administration of exogenous SP-A, as shown previously with proteomic studies of the SP-A rescued KO AM (7) and lead to an improved survival rate after infection (17, 18). Thus, SP-A is important for the organization of AM CMPs in response to infection and to a lesser degree at AM baseline conditions.

## Conclusion

In the absence of SP-A, a dysregulation may occur with regard to mechanisms that may lead to optimal outcomes, and this may be reflected in the number and composition of CMPs, as shown in the present study. Moreover, SP-A may contribute directly to CMP formation *via* interactions with various cell surface molecules, and in its absence these CMPs cannot be formed. The KO AM is not dormant and it still generates unique CMPs in both the baseline state and in response to infection, but these are not sufficient to enable a successful response in numerous readouts, as shown by published studies (19, 47). Although the mechanisms for CMP generation are not known, SP-A may play a direct or indirect role, as noted above. CMPs, in the presence and absence of SP-A at baseline conditions or in response to infection, consist of proteins whose levels may differ as well as proteins whose levels may not change. Although, the latter may be equally important in enabling the formation of protein patterns necessary to combat an insult, the present study was not designed to address this. However, the present study provides part of the foundation for further investigation. Future studies are needed to explore CMP differences by employing additional markers or antibodies, exogenous SP-A treatment (in WT mice) or rescue (in KO mice), or other, in order to experimentally manipulate specific markers that differ in WT vs KO AM CMPs. It is also possible that the binding of SP-A, either by itself or bound to pathogens, to cell surface molecules such as those studied here may play a role in the organization of CMPs that are important in host defense against infection. Such studies using technologies like TIS may identify key proteins responsible for the integrity of given CMPs necessary for host defense and other functions and help determine whether these potentially could serve as therapeutic agents or targets (52).

## Data Availability Statement

The raw data supporting the conclusions of this article will be made available by the authors, without undue reservation.

## Ethics Statement

The animal study was reviewed and approved by The Institutional Animal Care and Use Committee of the Penn State College of Medicine.

## Author Contributions

DSP conducted TIS experiments, processed data, interpreted results, and wrote the manuscript. VMC converted .xml data files to SAS, allowing more extensive analysis and performed the statistical analyses on the dataset. XZ prepared bacteria, treated mice with vehicle or bacteria, recovered AM from mice, and prepared slides for TIS. DS provided helpful suggestions for antibody calibration, operation of the TIS system, and use of TIS software for processing of data. JW provided guidance and suggestions for conducting TIS experiments and processing data and participated in planning of the manuscript. JF was responsible for overall direction of the project including: developing experimental design, interpreting results, and manuscript preparation. All authors contributed to the article and approved the submitted version except JW (who passed away prior to its completion).

## Funding

This work was supported by a grant from the National Institutes of Health (NIH 1R21AI113050-01A1) and by the G. Pedlow Research Fund.

## Conflict of Interest

The authors declare that the research was conducted in the absence of any commercial or financial relationships that could be construed as a potential conflict of interest.

## Publisher’s Note

All claims expressed in this article are solely those of the authors and do not necessarily represent those of their affiliated organizations, or those of the publisher, the editors and the reviewers. Any product that may be evaluated in this article, or claim that may be made by its manufacturer, is not guaranteed or endorsed by the publisher.

## Abbreviations

AM, alveolar macrophage; CMP, combinatorial molecular phenotype; FITC, fluorescein isothiocyanate; KO, SP-A knockout; KOI, knockout infected; KOV, knockout vehicle-treated; SP-A, surfactant protein-A; TIS, toponome imaging system; WI, wild type infected; WT, wild type; WV, wild type vehicle.
